# Information Patients With Melanoma Spontaneously Report About Health-Related Quality of Life on Web-Based Forums: Case Study

**DOI:** 10.2196/27497

**Published:** 2021-12-07

**Authors:** Rachel R J Kalf, Diana M J Delnoij, Bettina Ryll, Marcel L Bouvy, Wim G Goettsch

**Affiliations:** 1 Department of Pharmacoepidemiology and Clinical Pharmacology University Utrecht Utrecht Netherlands; 2 National Health Care Institute Diemen Netherlands; 3 Erasmus School of Health Policy and Management Erasmus University Rotterdam Rotterdam Netherlands; 4 Melanoma Patient Network Europe Uppsala Sweden

**Keywords:** reimbursement decision-making, QoL, health care, quality of life

## Abstract

**Background:**

There is a general agreement on the importance of health-related quality of life (HRQoL). This type of information is becoming increasingly important for the value assessment of health technology assessment agencies in evaluating the benefits of new health technologies, including medicines. However, HRQoL data are often limited, and additional sources that provide this type of information may be helpful.

**Objective:**

We aim to identify the HRQoL topics important to patients with melanoma based on web-based discussions on public social media forums.

**Methods:**

We identified 3 public web-based forums from the United States and the United Kingdom, namely the Melanoma Patient Information Page, the Melanoma International Forum, and MacMillan. Their posts were randomly selected and coded using qualitative methods until saturation was reached.

**Results:**

Of the posts assessed, 36.7% (150/409) of posts on Melanoma International Forum, 45.1% (198/439) on MacMillan, and 35.4% (128/362) on Melanoma Patient Information Page focused on HRQoL. The 2 themes most frequently mentioned were *mental health* and *(un)certainty*. The themes were constructed based on underlying and more detailed codes. Codes related to *fear, worry and anxiety*, *uncertainty*, and *unfavorable effects* were the most-often discussed ones.

**Conclusions:**

Web-based forums are a valuable source for identifying relevant HRQoL aspects in patients with a given disease. These aspects could be cross-referenced with existing tools and they might improve the content validity of patient-reported outcome measures, including HRQoL questionnaires. In addition, web-based forums may provide health technology assessment agencies with a more holistic understanding of the external aspects affecting patient HRQoL. These aspects might support the value assessment of new health technologies and could therefore help inform topic prioritization as well as the scoping phase before any value assessment.

## Introduction

### Background

Decisions on the reimbursement of innovative medicines in Europe are most prominently based on the recommendations of national health technology assessment (HTA) agencies. Conventionally, these HTA recommendations are prepared directly after market-authorization of medicines. The starting point for these HTAs is the assessment of (added) therapeutic value, also known as relative effectiveness assessment, and subsequently, cost-effectiveness assessments. In both, relative effectiveness assessments and cost-effectiveness assessments, outcome measures such as the overall survival rate, adverse events (AEs), and health-related quality of life (HRQoL) are considered.

From the perspective of patients, HRQoL is an important outcome measure because it can capture how disease and treatment affect a patient’s quality of life [1]. This is especially of interest in diseases such as cancer, where medicines may increase overall survival rates but may cause considerable toxicity. Therefore, HRQoL intends to inform HTA agencies on the relevance and added value of new oncology treatments for patients, for instance, if the medicine improves HRQoL by halting the progression of the disease, or alternatively, decreases HRQoL if toxicity or AEs have a large impact on the patient’s well-being.

Although the assessment of HRQoL is becoming increasingly important in different areas of health care, relevant HRQoL data are often unavailable. For instance, patients with severe disease seem less likely to complete HRQoL questionnaires compared with their healthier counterparts [2,3]. The use of complicated HRQoL instruments increases respondent burden and may also lead to lower completion rates. Furthermore, patients might not be motivated to complete HRQoL questionnaires in a research setting if tangible respondent benefits are not delivered. Overall, HRQoL data are currently only sparsely represented in HTA reports for oncological products. More specifically, only in a third of HTA assessments were HRQoL data used [4], leading to a low impact of HRQoL on HTA decision-making despite the general recognition of the importance of HRQoL for patients and society.

In addition to the limited availability of HRQoL data, current methods used to measure HRQoL may fail to truly capture what is most relevant to patients [5], which may result in incorrect overall interpretation. Therefore, there is a continuous search for sources that provide additional relevant information on HRQoL. Social media is a convenient and well-established communication source and therefore presents an obvious potential option. Patients often use social media to gather information on their health condition and treatment options, to share their experiences, and to find social support [6-8]. Previous research has also shown that social media may help identify HRQoL topics of importance to patients, prioritize the topics most relevant to patients, or help in the distribution of HRQoL questionnaires [9-12]. Melanoma is an area of oncology that has seen the rapid introduction of several classes of new therapeutics with new modes of action, increasing the likelihood of existing HRQoL tools failing to capture patient-relevant outcomes [13-15]. Concomitantly, several web-based patient forums for melanoma have been active.

### Objectives

To evaluate the potential relevance of social media as a meaningful source of HRQoL information for HTA, we identified the HRQoL topics that are most important to patients with melanoma based on discussions from web-based forums. Following the logic that in an unsupervised setting, patients would bring up topics relevant to them rather than being triggered by, for instance, a questionnaire, we focused on the research question: Which HRQoL topics do patients with melanoma and their caregivers spontaneously discuss on the web?

## Methods

### Overview

For this study, we focused on public web-based forums that are publicly accessible to anyone, as opposed to private patient communities. These public web-based forums provide peer support for a range of medical conditions, allowing the patients to share their experiences and provide information [16-18]. In a previous study, we collected patient perspectives on HRQoL from private social media sources, including a private Facebook (Facebook, Inc) group for patients with melanoma [9]. Using a different type of social media in this study allows for comparisons between the different sources of social media regarding the HRQoL topics discussed.

### Selection of Web-Based Forums

The public web-based forums were identified using 2 internet search engines, namely (1) Google (Google, Inc) and (2) Bing (Microsoft, Inc), which are currently the most popular search engines in the world [19,20]. Searches were conducted in English, with a combination of the search terms *melanoma*, *forum*, *message board*, and *discussion board*. Browser history was cleared before each search because the previous searches might influence the search findings. This forum search was conducted on 5 consecutive days starting June 4, 2019, to account for the websites being unavailable owing to maintenance issues. The search results from the first 2 pages shown by (1) Google and (2) Bing were extracted and assessed for eligibility, and any advertisements or images were excluded.

Forums were eligible for inclusion based on the following three criteria: (1) the website had been active for ≥5 years based on the publication dates of posts, (2) at least 2000 posts had been posted on the forum, and (3) ≥5 new posts had been posted in the past week. We identified 3 forums as eligible for inclusion: Melanoma Patients Information Page (MPIP), Melanoma International Forum (MIF), and MacMillan Cancer Support Online Community for Melanoma Patients. Each forum was informed of our intention to use their publicly available posts for research purposes via email. Both MPIP and MIF are forums based in the United States and MacMillan is based in the United Kingdom. MPIP and MIF focus solely on patients with melanoma, whereas MacMillan provides information to support patients with cancer in general, in addition to having 64 cancer-specific forums (eg, melanoma, Hodgkin lymphoma, pancreatic cancer, and unknown primary cancer) [21-23].

### Data Extraction

No login was used to gain access to any of the posts extracted, nor was login required on any of the forums. Each forum thread was sorted by the date of the last post, after which all the threads were collected. A thread is defined as a collection of posts, with an initial post that introduces a specific topic and the subsequent replies posted by one or more members of the forum. We collected the complete threads from each forum using the R package *rvest* (R Core Team) in September 2019 [24]. We collected the following data: title of the thread, text from each post, username of each post, date and time of each post, and whether a post was the original post or a reply. Each username was given a user ID to ensure anonymity.

### Data Analysis

We coded the posts using the coding scheme developed in our previous study [9], in which members of the Melanoma Patient Network Europe, an established patient network for patients with melanoma, caregivers, and advocates, were approached via its multiple social media channels to anonymously complete a 25-item web-based survey. In this survey, questions regarding sociodemographic and clinical characteristics and several open questions exploring patient and caregiver perspectives on HRQoL (eg, “What is HRQoL in melanoma for you?”, “Name 3 things that deteriorate your/the melanoma patient’s HRQoL today?”) were posed. Two researchers independently performed inductive content analysis on the responses to the open-ended questions and assigned codes, and any discrepancies in coding were resolved by consensus. As these themes and codes may not have covered all the topics spontaneously discussed in the forums, we created new themes and codes as required. The following themes were added: *alone* and *coping*, and the code *guilt* was added to the theme *certainty*. In addition, we adjusted the coding scheme to be more concise.

We excluded the posts that did not focus on HRQoL or melanoma, provided advice or shared experience, asked a question or provided information, or offered support. We defined HRQoL as the patient’s subjective perception of the impact of the disease and its treatment on the physical, psychological, and social aspects of daily life [25,26]. From each forum, a random sample of 100 posts was coded by 4 authors (RRJK, DMJD, WGG and MLB). Agreement regarding the inclusion and exclusion of posts between the coders was 74% for MPIP (RRJK and DMJD), 85% for MIF (RRJK and WGG), and 83% for MacMillan (RRJK and MLB); any disagreements were discussed and resolved by consensus. From this random sample, 44% (44/100) were included in this study from MPIP, 61% (61/100) from MIF, and 63% (63/100) from MacMillan. Subsequently, author RRJK continued coding the posts selected randomly from each forum until 100 posts which referred to HRQoL aspects were included. After this, the posts were coded in batches of 25 until saturation. We defined saturation as not being able to identify a new emerging theme in 2 consecutive batches of 25 posts [27]. Owing to the vast number of posts in each forum, we decided to code until saturation because this was sufficient to identify which HRQoL aspects were relevant to patients with melanoma. When author RRJK was unsure about a specific post or code, the issue was discussed and resolved by consensus among authors (RRJK, DMJD, MLB, and WGG). A total of 72.4% (262/362) posts for MPIP, 75.6% (309/409) for MIF, and 77.2% (339/439) for MacMillan were assessed solely by author RRJK. Coder drift was not assessed in this study, and therefore, poses a potential coding bias.

Covering all the posts assessed, we conducted an analysis of the number of threads and reply posts by each unique user to assess how often the same person initiated a thread or replied to an initial post. As a subanalysis, we assessed the subforums available on MIF in more detail. MIF provides separate forums for patients with melanoma with stage I and II, stage III and stage IV, as well as separate forums for newly diagnosed (ND) stage I and II patients and ND stage III and IV patients. This allowed us to evaluate which HRQoL topics were important for patients with melanoma at different disease stages. The results from analyzing the forum posts have been described qualitatively. This study was conducted in accordance with the Standards for Reporting Qualitative Research [28]. All data were collected, coded, stored, and analyzed using R version 3.4.4 (R Core Team) and NVivo (version 12; QSR International) [29,30].

## Results

### Overview

**Figure 1:**
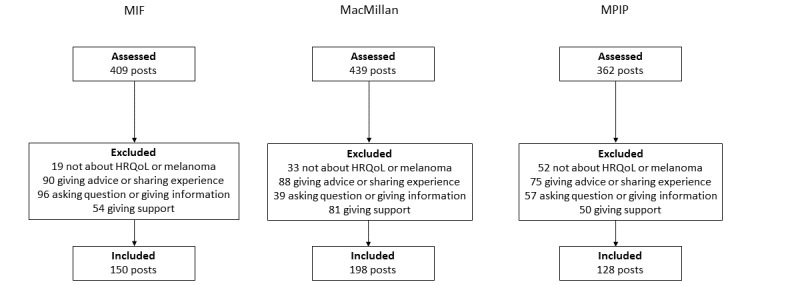
Overview of inclusion and exclusion criteria for forum posts. HRQoL: health-related quality of life; MIF: Melanoma International Forum; MPIP: Melanoma Patients’ Information Page.

A total of 14,755, 6798, and 1671 threads were collected from MPIP, MIF, and MacMillan, respectively. This resulted in 88,261, 23,911, and 9551 original posts from MPIP, MIF, and MacMillan, respectively. A total of 409 posts from 189 unique users were assessed from MIF, as were 439 posts from 359 unique users from MacMillan and 362 posts from 243 unique users from MPIP (Figure 1). After the exclusion of irrelevant posts, 150 posts from 112 unique users, 198 posts from 164 unique users, and 128 posts from 96 unique users were included in our assessment from MIF, MacMillan, and MPIP, respectively. We determined how often the same user started a thread and posted a reply (Figure S1 in Multimedia Appendix 1). Some users started 1 thread but did not reply to any other post (46/189, 24.3% on MIF; 130/359, 36.2% on MacMillan; and 71/243, 29.2% on MPIP). Another group of users posted 1 reply, but did not start any threads (99/189, 52.4% MIF; 67/359, 18.7% MacMillan; and 52/243, 21.4% MPIP). Finally, a number of users started 1 thread and posted 1 reply (9/189, 4.8% MIF; 69/359, 19.2% MacMillan; and 32/243, 13.2% MPIP). Overall, 95.8% (181/189) of the users on MIF, 95% (341/359) on MacMillan, and 92.6% (225/243) on MPIP posted ≤5 posts (either as the initial thread or reply post). Only a few users in each forum contributed to a greater extent. Only 2 major outliers can be identified: 1 on MPIP, where 1 user started 45 threads and posted 26 replies, and 1 on MIF, where 1 user started 20 threads and posted 74 replies.

On all 3 forums, the 2 most often identified themes were *mental health* and *certainty* (Table 1). More than half of the posts mentioned aspects related to *mental health* (85/150, 56.7% MIF; 126/198, 63.6% MacMillan; and 69/128, 53.9% MPIP), and at least a third of the posts mentioned information relevant to *certainty* (63/150, 42% MIF; 80/198, 40.4% MacMillan; and 40/128, 31.3% MPIP). Other often-mentioned themes were *health care communication* (32/150, 21.3%) and *unfavorable effects* (28/150, 18.7%) on MIF, *health care access* (43/198, 21.7%) and *unfavorable effects* (27/198, 13.6%) on MacMillan, and *health care access* (20/128, 15.6%) and *unfavorable effects* (21/128, 16.4%) on MPIP.

**Table 1:** Total number and percentage of posts mentioning a specific theme on each forum (N=476).

Theme	Total posts per forum, n (%)		
	MIF^a^ (n=150)	MacMillan (n=198)	MPIP^b^ (n=128)
Mental health	85 (56.6)	126 (63.6)	69 (53.9)
Certainty	63 (42)	80 (40.4)	40 (31.2)
Health care communication	32 (21.3)	21 (10.6)	12 (9.4)
Unfavorable effects	28 (18.6)	27 (13.6)	21 (16.4)
Health care access	16 (10.6)	43 (21.7)	20 (15.6)
Health care general	17 (11.3)	23 (11.6)	18 (14.1)
Disease status	16 (10.6)	5 (2.5)	9 (7)
Support	15 (10)	26 (13.1)	12 (9.4)
Coping	14 (9.3)	22 (11.1)	4 (3.1)
Social life	14 (9.3)	19 (9.6)	17 (13.3)
Health general	13 (8.7)	7 (3.5)	6 (4.7)
Physical health	9 (6)	11 (5.6)	8 (6.3)
Treatment	9 (6)	4 (2)	14 (10.9)
Happiness	8 (5.3)	7 (3.5)	6 (4.7)
Alone	1 (0.7)	3 (1.5)	1 (0.8)

^a^MIF: Melanoma International Forum.

^b^MPIP: Melanoma Patients Information Page.

Each theme was constructed from underlying, more detailed codes. Table S1 in Multimedia Appendix 1 shows the codes used for each forum and provides excerpts from posts to provide examples for each code. This provides insight into the construct of each code and displays in more detail which HRQoL aspects the patients spontaneously discussed on the web. Examples of the most-often discussed codes (Table 2) are given below.

**Table 2:** Total number and percentage of posts mentioning a specific code on each forum (N=476).

Theme and code			Total posts per forum, n (%)				
			MIF^a^ (n=150)		MacMillan (n=198)		MPIP^b^ (n=128)
**Mental health**							
	Fear, worry, and anxiety^c^	58 (38.8)		78 (39.4)		46 (35.9)	
	Positive mood	11 (7.3)		17 (8.6)		10 (7.8)	
	Mental health^d^	3 (2)		16 (8.1)		2 (1.6)	
	No anxiety or relieve	5 (3.3)		7 (3.5)		4 (3.1)	
	Stress	6 (4)		4 (2)		4 (3.1)	
	Not to worry	2 (1.3)		2 (1)		3 (2.3)	
	Depression	0 (0)		2 (1)		0 (0)	
**Certainty**							
	Uncertainty	38 (25.3)		46 (23.2)		20 (15.6)	
	Hope	13 (8.7)		18 (9.1)		13 (10.2)	
	Confusion	5 (3.3)		11 (5.6)		4 (3.1)	
	Guilt^d^	4 (2.7)		5 (2.5)		1 (0.8)	
	Confident	1 (0.7)		0 (0)		2 (1.6)	
	Control	2 (1.3)		0 (0)		0 (0)	
**Health care communication**							
	Lack of information	14 (9.3)		10 (5.1)		4 (3.1)	
	Informed decision-making	11 (7.3)		5 (2.5)		4 (3.1)	
	Good information	6 (4)		1 (0.5)		3 (2.3)	
	Counselling	1 (0.7)		2 (1)		1 (0.8)	
	Access to information	0 (0)		3 (1.5)		0 (0)	
**Unfavorable effect**							
	Unfavorable effects	25 (16.7)		24 (12.1)		19 (14.8)	
	No unfavorable effects	3 (2)		3 (1.5)		2 (1.6)	
**Health care access**							
	Waiting time	4 (2.7)		29 (14.6)		10 (7.8)	
	Finances	6 (4)		7 (3.5)		5 (3.9)	
	Access medicines	5 (3.3)		4 (2)		1 (0.8)	
	Access care	1 (0.7)		3 (1.5)		4 (3.1)	
**Health care general**							
	Good care or good doctors^c^	14 (9.3)		10 (5.1)		14 (10.9)	
	Bad care or bad doctors	3 (2)		13 (6.6)		4 (3.1)	
**Disease status**							
	No spreading	5 (3.3)		4 (2)		1 (0.8)	
	No evidence of disease	7 (4.7)		0 (0)		5 (3.9)	
	Progression	2 (1.3)		1 (0.5)		3 (2.3)	
	Metastasis	2 (1.3)		0 (0)		0 (0)	
**Support**							
	Support^d^	14 (9.3)		14 (7.1)		9 (7)	
	Ignorance	1 (0.7)		6 (3)		1 (0.8)	
	Lack of support	0 (0)		6 (3)		2 (1.6)	
Coping^d^			14 (9.3)		22 (11.1)		4 (3.1)
**Social life**							
	Patient network	12 (8)		11 (5.6)		15 (11.7)	
	Work	2 (1.3)		3 (1.5)		1 (0.8)	
	Friends	0 (0)		2 (1)		1 (0.8)	
	Family	0 (0)		3 (1.5)		0 (0)	
**General health**							
	Pain	9 (6)		3 (1.5)		3 (2.3)	
	Diet and appetite	3 (2)		1 (0.5)		3 (2.3)	
	Good health	1 (0.7)		1 (0.5)		0 (0)	
	Pain free	0 (0)		2 (1)		0 (0)	
**Physical health**							
	Fatigue	5 (3.3)		7 (3.5)		5 (3.9)	
	Good physically	1 (0.7)		2 (1)		1 (0.8)	
	Pregnancy^d^	1 (0.7)		1 (0.5)		2 (1.6)	
	Exercise	2 (1.3)		1 (0.5)		0 (0)	
**Treatment**							
	Randomized controlled trials	5 (3.3)		1 (0.5)		6 (4.7)	
	Good medicines	1 (0.7)		3 (1.5)		7 (5.5)	
	Drug effectiveness	3 (2)		0 (0)		1 (0.8)	
**Happiness**							
	Enjoy life	4 (2.7)		5 (2.5)		1 (0.8)	
	Normal life	3 (2)		2 (1)		5 (3.9)	
	Capability	1 (0.7)		0 (0)		0 (0)	
Alone^d^			1 (0.7)		3 (1.5)		1 (0.8)

^a^MIF: Melanoma International Forum.

^b^MPIP: Melanoma Patients Information Page.

^c^Codes combined as compared with coding scheme used in previous study.

^d^New codes added to the original coding scheme used in previous study [9].

### Fear, Worry, and Anxiety

On all 3 forums, the code relating to *fear, worry, anxiety* was most often discussed (Table 2; Table S1 in Multimedia Appendix 1). More specifically, on all forums, users talked about being obsessed over moles and being scared about their diagnosis. Other aspects mentioned included, but were not limited to, being anxious about the results (MIF and MPIP), worrying about recurrences (MIF), and the consequences of stopping treatment (MIF and MPIP).

### Uncertainty

The second most frequently discussed topic on MIF, MacMillan, and MPIP was *uncertainty* (Table 2). Users were uncertain about many different aspects, such as whether they had made the right decision, whether the medicines would work, if the diagnosis was correct, and how bad the AEs would be (Table S1 in Multimedia Appendix 1). For example, one user said “[...] any suggestions on [...] how not to worry endlessly about the ‘what ifs’.”

### Unfavorable Effects

On MPIP, MacMillan, and MIF the topic *unfavorable effects* was also discussed commonly (Table 2). This focused on the AEs, the complications and the symptoms that the patients experienced. One specific AE that was most frequently mentioned on MIF and MacMillan was lymphedema (Table S1 in Multimedia Appendix 1). Users also discussed solutions to the AEs and the complications they were experiencing, such as those from the medicines they were prescribed (MIF, MacMillan, MPIP). Not only were the intolerable AEs, complications, and symptoms discussed, but also those that were manageable. Discussions reflected the different degrees of AE presentation experienced by patients, from manageable to intolerable. For example, 1 user mentioned the following “not the end of the world itching and rash, but it is very maddening and crazy making*.*”, while another indicated “[...] has a terrible rash on his face head and back. We can LIVE with the rash*.*”

### Waiting Time and Coping

On MacMillan, next to *unfavorable effects*, both *waiting time* and *coping* were often mentioned (Table 2). Coping was also a topic discussed on the other 2 forums (Table S1 in Multimedia Appendix 1), although it seemed to be discussed to a lesser extent. Users discussed how they coped, for example, with their diagnoses (MIF and MacMillan), with the AEs (MIF and MacMillan), and with their lives in the new normal (MIF and MPIP). Some users indicated how difficult it was to cope with their diagnosis and how they went through denial before being able to accept the seriousness of it all (MacMillan). The long waiting time for appointments and results were also mentioned on all forums (Table S1 in Multimedia Appendix 1). Users expressed this as: *feels like waiting for eternity*, and *the waiting game being the worst*. However, some users on MacMillan also indicated that the waiting time was not as long as anticipated.

### Hope

Hope was also a code mentioned in all forums. On MIF, a user expressed the following: “I’m getting to the point where I’m believing I could be ok again!” Users also expressed their hope of having scans that showed tumor shrinkage (MacMillan and MPIP) and their hope for medicines that would work (MacMillan and MPIP).

### Health Care

Members shared experiences related not only to their health, but also to their experiences with health care, including access to health care, lack of information, and making informed decisions (Table S1 in Multimedia Appendix 1). On all forums, users talked about good and bad experiences with their health care. For example, one user posted:

I was seen by a new (?), certainly very young doctor who had obviously not read my notes as he had no idea that I was on the Avastin trial. In fact he didn't even know what the trial was and even asked me to spell the drug's name for him!!!! Obviously a very well read young man in his specialist field, not!

However, good experiences with health care were also shared, such as by this user:

Had my first PET this week since stage 4 dx, and met with Onc the same day to go over results. She hadn't looked at them yet when we met, so I was pretty nervous. She could tell and just told me these are the first scans and the only bad results would be if there are any new mets that had popped up in kidney, lungs, or any other organs. She said she would be happy with no change, or even if things only grew by a little.

The subanalysis of MIF subforums (data not shown) showed that fear, worry and anxiety was discussed on all subforums, but most often by patients with stage I or II, with 55.0% (33/60; including ND) of the posts mentioning this topic. Uncertainty was discussed on all subforums to approximately the same extent (17.6% (6/34) - 32.3% (10/31) of the posts discussed this topic). The topic unfavorable effects was more often discussed by stage III and IV patients (25.6% (22/86) including ND) than by stage I and II patients (5.0% (3/60) including ND). ND patients discussed coping more often than patients who were not posting on the ND subforums (17.2% (11/64) vs 3.7% (3/82), respectively).

## Discussion

### Principal Findings

In this study, we showed that patients with melanoma and their caregivers discussed many different topics related to HRQoL on public web-based forums. Topics related to *fear, worry and anxiety*, *uncertainty*, and *unfavorable effects* were most often discussed. With respect to *fear, worry and anxiety*, some users discussed their worries regarding their moles and diagnosis, which may be most important to patients in the earlier stages of melanoma. Other users discussed aspects related to their fear of recurrence or the consequences of stopping treatment, which may be more relevant to patients in the later stages of the disease. Of note, a caveat of social media is the incomplete information on user characteristics, making it infeasible to determine the disease stage for each user. Many users also discussed aspects related to *uncertainty*. However, this covered different aspects ranging from uncertainty regarding AEs and the effectiveness of the medicines to uncertainty about their diagnosis. Finally, with respect to discussions on unfavorable effects, users shared their experiences with AEs and complications, as well as their solutions.

It is important to realize that the type of social media used may affect the results of a study like ours because social media may be public (anyone may gain access to posts without signing in) or private (where an account is needed before users may gain access to posts). In public sources, users may be less inclined to share personal experiences as compared with private social media sources [31,32]. Previously, we had assessed which HRQoL topics were most important to patients with melanoma and their caregivers on private social media by posting a survey on the private social media channels of Melanoma Patient Network Europe [9]. It was shown that *family* and having a *normal life* were the 2 most important HRQoL topics for patients with melanoma. In this study, patients with melanoma most often discussed topics related to *fear, worry, anxiety*, *uncertainty*, and *unfavorable effects*. This difference may be because in the previous paper, we actively inquired about the HRQoL aspects most important to patients with melanoma, guiding them through a survey, whereas in the current paper, we merely listened to the topics that patients with melanoma discussed with each other [33,34], the latter being a much more inductive approach.

Another aspect that may influence our study results is the overrepresentation of a specific group of users, such as patients with a specific stage of disease or their caregivers discussing the topics most important to them and subsequently driving our results. We previously showed using private social media that patients with melanoma with a different stage of the disease find other HRQoL aspects important, as do caregivers [9]. In this study, we confirmed this as our subanalysis of the MIF subforums suggested that different HRQoL topics seemed important to patients with melanoma in different disease stages. Subsequently, melanoma-specific HRQoL questionnaires may benefit from taking these differences into account.

Previous research has shown that disease-specific HRQoL questionnaires do not fully represent what patients find important in HRQoL [9,12,35]. For example, the wording in the questionnaires may be different from how patients describe HRQoL aspects; some topics may seem less relevant to patients and some topics may not be included in the HRQoL questionnaires [9,12,35]. Therefore, we evaluated whether melanoma-specific HRQoL questionnaires represented topics discussed by patients with melanoma on web-based forums. In both the Functional Assessment of Cancer Therapy-Melanoma (FACT-M) and European Organization for Research and Treatment of Cancer (EORTC) QLQ-MEL38, some questions related to the theme *mental health* are present [36,37]. In FACT-M, these questions seem to focus on worrying, losing hope, being sad, and feeling nervous. Although in EORTC QLQ-MEL38, they seem to focus mainly on worrying, including worrying about unfavorable effects. In contrast, web-based discussions seem to focus more on the (overwhelming) fear and anxiety of patients with melanoma. Regarding *uncertainty*, only EORTC QLQ-MEL38 poses one question *Have you felt able to plan for the future?* However, in web-based discussions, other aspects of uncertainty seem to be more important to patients. Several questions related to *unfavorable effects* are posed in both FACT-M and EORTC QLQ-MEL38, including some questions related to lymphedema. This seems to correspond to the web-based discussions among patients with melanoma. Other themes that were often discussed on the web included *health care communication* and *health care access*. It seems that only EORTC QLQ-MEL38 has questions focusing on these themes. Although these melanoma-specific HRQoL questionnaires have been developed with great care, these findings raise questions about the extent to which these questionnaires cover aspects most pertinent to patients. Therefore, HRQoL questionnaires may benefit from ensuring that topics correspond more to patient experiences, such as including more questions on uncertainty.

It is important to note that although aspects related to AEs may be important for reimbursement decision-making, aspects related to uncertainty and coping are less relevant. However, considering the high psychological burden in the early stages of melanoma, which contrasts with the seemingly benign overall survival outcomes, some topics may become increasingly relevant for HTA as melanoma therapies move from the advanced setting into earlier stages of the disease. These insights highlight the importance as well as the burden that these topics present for patients with melanoma across all disease stages, in addition to disease-specific concerns. Health care systems, therefore, should be aware that topics such as health care communication and access to services can critically impact the HRQoL of patients, irrespective of the given treatment.

### Limitations

This study has several limitations. First, we focused on web-based forums, whereas other public, social media channels might provide a different type of insight (eg, Twitter, public Facebook groups, or blogs). However, not every social media channel is appropriate for gathering insights on a specific topic. For example, information on AEs is not readily available on Facebook or Twitter [38]. Second, identifying the disease stage for each patient was difficult but has been proven to be relevant as our earlier analysis of stage-specific forums has shown. This could possibly be overcome to a certain degree by using more automated methods of data analysis. In addition, validating authenticity (eg, verifying whether users actually have the disease they discuss) on the web is difficult [11,39,40]. Third, selection bias may be an issue because the patient population present on web-based forums may be different from the patient population that is not using web-based forums. For example, patients using social media are conventionally better educated [40,41], more likely to be female [39,42], and may have a different symptom experience [43]. Finally, web-based forums may update their terms of use at any given time. At the time of collecting the posts, all 3 forums were of public nature and logging in to gain access to the posts was not necessary. However, MIF has changed this and now requires a login to gain access to posts.

### Strengths

One of the strengths of this study was the coding of 100 posts from each forum by 2 authors to ensure validity. Analyzing qualitative data can be subjective; therefore, agreement among multiple authors when assigning codes is important. In addition, any uncertainties in the posts that were coded until saturation was reached were discussed and resolved by consensus among the 4 authors to further ensure validity. Another strength was determining how often users posted an initial post and a reply post to assess whether one or more users could possibly drive our results. A total of 94.4% (747/791)of users posted only a few posts (≤5 posts) on the forums, suggesting that our results were not driven by one or more users. It seems highly unlikely that the 2 outliers in MIF and MPIP would drive the results, considering the number of posts assessed.

### Implications and Conclusions

Patient involvement is becoming increasingly important in HTA, which is especially appreciated during the scoping phase of HTA and for HTA topic prioritization [44,45]. The scoping phase is conducted at the beginning of an HTA assessment, where the technology under assessment, the reference or comparator technology, the relevant study population, and the relevant outcome measures regarding the effectiveness and safety of the technology under assessment are identified [46,47]. In an ideal situation, several stakeholders, including clinicians and patients, should be involved during this scoping phase. The input from patients or their representatives is, for example, important to choose outcome measures that matter to patients. However, the involvement of patients or representatives may be limited [48-51]. Social media may allow inputs from a wide group of patients, and may thus provide robust insight into patient experiences. For example, in the scoping phase, social media may be informative in determining which outcome measures would be most important to measure. Social media may not only prove useful in HTA but may also inform health care professionals in their understanding of patient experiences and what is important to patients regarding their treatment and health care [52]. In addition, issues relevant to patients and which they deal with on a regular basis may be uncovered and could lead to identifying issues that might have otherwise gone unrecognized [53]. In addition, in regulatory decision-making, information from social media may help determine which AEs greatly affect HRQoL, are most debilitating to patients, and which AEs are acceptable to patients [39,54,55]. Therefore, social media may be informative for several stakeholders with varying goals. However, this source of information still needs to become part of the regular data extraction practices of stakeholders. Therefore, clear guidelines are needed for the ethical use of social media data, the limitations that are involved, and the purposes for which this information could be used.

To conclude, it is important to realize that web-based forums are a valuable source to cross-reference the relevance of existing tools and help identify gaps in existing procedures. Social media may contribute to improving the content validity of patient-reported outcome measures, including HRQoL measures. More specifically, current melanoma HRQoL questionnaires may potentially improve patient relevance by adding more items related to fear, worry, anxiety and uncertainty. Social media is a readily available source that can provide fast inputs from patients with both rare and common diseases. It can be used passively to listen to what patients discuss on the web and to actively distribute questionnaires. In addition, information extracted from social media may support an evidence ecosystem, where existing evidence is used by several stakeholders for different goals. This information source may contribute to a more holistic understanding of the patient’s perspective and highlight external factors affecting patient HRQoL. Social media may specifically provide insights for HTA decision-making during the prioritization of topics as well as during the scoping phase conducted before the value assessment of a new health technology.

## Appendix

Multimedia Appendix 1 An overview of the codes per forum including examples illustrating each code, and the number of threads and reply posts each unique user posted on each forum.

## Abbreviations

AE: adverse event
EORTC: European Organization for Research and Treatment of Cancer
FACT-M: Functional Assessment of Cancer Therapy-Melanoma
HRQoL: health-related quality of life
HTA: health technology assessment
MIF: Melanoma International Forum
MPIP: Melanoma Patients’ Information Page
ND: newly diagnosed
