# Supplementary material for: Information Patients With Melanoma Spontaneously Report About Health-Related Quality of Life on Web-Based Forums: Case Study
Source: J Med Internet Res. 2021 Dec 7;23(12):e27497. doi: 10.2196/27497 (PMC8693198; doi:10.2196/27497)

Table S1 Overview of codes per forum and examples to illustrate each code

Code	Forum		
	MIF	MacMillan	MPIP
**Unfavourable effects**			
Unfavourable effects	'Sorry your lymphedema is still so bad. I'm still struggling with it, but have gotten to a manageable place with it.'	'I found that lymphedema was way down the consultants' list of concerns…although it was a really big deal for me.'	'But I hit a wall after my 4th infusion. It has kicked my tail with nausea, fatigue, some diahrea, just a bad general overall feeling. So a month after my last infusion, I am still battling side effects. At least I was able to finish my 4 treatments, but feeling pretty rough the last month.'
No unfavourable effects	'She did the same treatment route that you have and like yourself she has not really had too hard of a time with the interferon. Other than being tired, she is pretty good most of the time.'	'So far I haven't had any side effects but monitor myself very carefully, and of course my wife watches me like hawk looking for any changes that I might realise are happening.'	'I didn't have much in the way of side effects through 3 treatments. Fatigue, nausea, stomach cramps and some in transit cancer popping out. For the most part, things went smooth.'
**Alone**	'unfortunate, but still nice to know I'm not alone.'	'I thought this was the best place to look…everyone who comes here is REAL!!!! No medical journals/papers just real people, which often helps me to feel not as alone!!'	'reminding me so quickly why we do this and we are not alone and we are all fighting together and this thing can be beaten.'
**Certainty**			
Confident	'[…] so hopefully everything is still clear and good.'	NA	'I'm 32 with a 2 month old baby who was born two weeks before I found the mass in my brain and two smaller spots in my lungs...so, I am not going down without the fight of a lifetime.'
Confusion	'This who thing [has] been a confusing, scary time not only for me but my wife and 2 young daughters.'	'I'm so confused and feel like I've been left in limbo'	'We haven't gotten a second opinion - should we? and if so where? Who?What questions should we be asking right now?We are trying to move past shocked and denial stages to take action.'
Control	'It is so hard to keep emotions under control.'	NA	NA

Guilt[1]	'As for tanning beds, yes, I started going at age 15, stupid! I went every winter 1-2 x a week for years.'	'I feel guilty complaining as I know there are others suffering more than me…'	'And then there will be the lingering fear of the unknown and the guilt that so many special people have died of this disease including […] and […] and so many others. I volunteer at a nonprofit that offers free support for cancer patients and their families. It helps.'
Hope	'My derm said that we wouldn't go back in and cut deeper unless we find something moderately dysplastic or worde. Yay!!! I'll be seeing my oncologist on the 15th of Jan for a standard follow-up, and feel certain that EVERY LIL THING, GOIN' BE ALRIGHT.'	'Fingers crossed tomorrow's scans are clear.'	'but, like i just said IM STILL HERE! that alone i keep telling myself haha...Its all good, im in the fight & hopefull...'
Uncertainty	'So I am going through anxiety again…the waves of fear, and what ifs, and when and how…'	'I have been told that I have a 50:50 chance of recurrence. I feel as though I spend my whole time pre-occupied watching and waiting for 'it' to happen.'	'I'd say the big thing with nivo is uncertainty. Is my knee pain because I'm getting older and did too much or a side effect? Is my stomach upset due to stress or a side effect? The joint pain is probably cause I overdid it.'
**Coping**	'I think that I am still in the process of accepting the diagnosis and trying to make adjustments (like sun avoidance btw. 10-4pam) and take them in stride.'	'I apologise if this is selfish but for the last 10 months I have been finding it difficult to cope. I put on a smiley brave face with my husband and children, am back at work but just cannot face discussing my concerns directly with anyone.'	'We are trying to move past shocked and denial stages to take action.'
**Disease Status**			
Metastasis	'[…] Frozen all came back good, until 2 days later they called me in and said I had micro mets to the left one and so I had surgery again that week, with removal of 10 nodes…all negative.'	NA	NA
No evidence of disease	'But I am alive and I am NED for now.'	NA	'The oncologist was happy to announce she considers me NED!'

No spreading	‘As usual the moles I was nervous about were normal. I have to remember that change isn’t necessarily cancerous. For me it’s normal to have the pigmentation on my moles change a bit.’	‘I got my results on Thursday and everything has stayed the same as the last scans 8 weeks ago, although there has been no shrinkage, there has been no growth either, so great news!’	‘I just got results from my most recent scan, and I am celebrating a full year since my last surgery which cleaned house of my major tumors. All clear, with no new progression.’
Progression	‘Well just back from Don’s colonoscopy & even though oncologist after reviewing PET was pretty hopeful would not be melanoma …. It is back. He had 14 montsh NED after IPI.’	‘I have a stage 4 melenoma, about 3 weeks ago mine was found to have spread into one of my lungs!	‘I had been on Keytruda for 3 months then liver progression, then TAF/MEK (short stay due to side effects), now I feel another in transit in my scar. These in transits seem to keep popping up in my same scar area even though I have had 2 WLE and CLND. Immunotherapy and Targeted Therapy don't seem to be working either. I am getting discouraged trying treatments only to see more mets and progression. I have a CT Scan and appt next week, need some positivity from long term Stage 4 warriors on this site as I am becoming discouraged with what seems like a downhill battle for me.’
**Happiness**			
Capability	‘The idea of not being able to go running again ever in my life if painful.’	NA	NA
Enjoy Life	‘For most of us it’s a huge wake up call to what is really important in life and you learn not to take anything for granted.’	‘I can live with the groovy compression sleeve but don't want to have to give up all the things that make me happy.’	‘I felt so incredibly lucky today. I was able to piddle about in my yard with the love of my life, feel the warming breeze on my face and see one more spring. Melanoma is more than able to take that moment, this day, from any of us. So as I tried to soak in the spring beauty that life afforded me once again, I thought about things.’
Normal Life	‘Just on the downside now and I can’t wait to feel normal.’	‘I still have my moments when I worry about the future and the usual paranoia over swollen glands etc, but generally I am back to normal.’	‘He is currently NED, we hope he stays that way forever! He works, goes hiking, does everything without any problems, just like before’

**Healthcare Access**			
Access Care	‘Also, before we started the trial we contacted MD Anderson, sent them all the scan results, blood work, etc. for second opinion and they said they can treat Dave there but we have to relocate to Huston and pay for everything.’	‘It seems in my area anyway that there are a lack of services / resources for this condition.’	‘In all my research, it seems that the nursing facilities in commuting distance for my mom don't have much information on how they help cancer patients. It has also been extremely challenging to find a nursing home willing to administer the targeted therapy medication my dad needs (they are taken orally, but due to his difficulty swallowing, they have been putting them through a feeding tube).’
Access Medicines	‘In the meantime I tried to join ONCOVex but it was a no go because I was not an UK citizen!’	‘I really hope that we can get this decision reviewed & Yervoy accepted - it's the only hope we have & it's cruel to deny us our lives - particularly younger sufferers & their dependant families. The cost of the drug must surely be outweighed by the cost to the system - when a young parent loses their life to the disease & the family can't afford to live without support from the state.’	‘At the time my only option was interferon or watch and wait. ‘
Finances	‘Though I am B-RAF positive and accepted from the phase 3 trial my insurance is causing trouble because it is just a trial … So I am fighting but preparing a plan B, C and D!’	‘it is awful worrying im considering trying to go private if i can afford it to be seen quicker and get a diagnosis back as me and my family are numb.’	‘No, my insurance denied the $8,500 bill. I don’t know if I’ll get billed by [hospital]. I was not made aware of the cost prior to the test being done.’
Waiting Time	‘I am going to [hospital] for this. The wait to get into [hospital] has been about a month, which has me very anxious.’	‘Think my anxiety is through the roof having waited 7 weeks to get the result.’	‘Oh gosh-isn't waiting the worst! I'm playing that waiting game right now and every time the phone rings I take a deep breath...you know what I mean!’

**Healthcare Communication**			
Access to information	NA	‘They sent out a letter for a follow up appointment on 09/11/2017.Yesterday I received a letter bringing forward my appointment to 03/11/2017. This immediately sent my mind in to overdrive so I called the Dermatologist clinic who told me they had results but couldn't tell me over the phone. Given that I live 100 miles away from the hospital I contacted my GP and explained.I received a call back from my GP an hour later, telling me I've got Melanoma. He was unable to tell me any more than that.I'm now worried sick, my whole world feels like it's being turned upside down.’	NA
Counselling	‘I’m not a particularly modes person (being a former actor will do that), but I’m 53, male, and a tad (hee) overweight, so standing there naked for my early -30s very attractive female derm is a tad offputting, BUT, it beats dying of cancer!’	‘I had severe depression a few months after my diagnosis last year. My GP referred me to a counsellor, I was sceptical at first but I went as I decided that it was worth a try. I can honestly say, it helped a great deal. My counselling finished around Christmas time and I am now feeling 100% better.’	‘I think I will discuss with her my thoughts/worries. Hoping she can take her 'dr' hat off long enough to put herself in my shoes and think about what she would want done.’
Good information	‘Doctors do not talk about the little things that can make a hospital stay so miserable, like your stool. I now feel much better prepared for my dissection.’	‘Thanks for the checkup and excision info - I got told a bit about what would happen at an excision when I had the biopsy, but also got told "but that won't happen" so didn't pay as much attention as I should have, in hindsight.’	‘Given my diagnosis I very much look to be informed so as to effectively manage my care ‘

Informed decision making	‘He is an amazing man. He was very positive and encouraging. He was actually encouraging no surgery – but my husband and I still felts that is was the best coarse for use – and [Doctor] was ok with that too.’	‘They reiterated that the SLNB was completely optional and up to me - I very nearly changed my mind but decided to go ahead in the end.‘	‘At the time, I think it was standard of care to recommend a SLNB for any lesion greater than 1mm. I was staged at that point, 3A, due to the two positive lymph nodes. After the SLNB, I transferred to [hospital] for my care. I discussed the CLND with my surgeon, [doctor] who reviewed the procedure versus watch and wait, I opted for the,procedure with no additional positive nodes. The only other testing was for BRAF as I recall.’
Lack of information	‘[…] Did anyone met doctors like mine? I feel they are ignorant and not serious. They don’t seem to put any effort in communicating with me and even when I ask educated questions, they brush me off like it’s nothing. Oh, and about the mole on my earlobe. I asked the surgeon about it and he said Oh, of course it’s melanoma. It’s just that we are not doing anything about it right now. We should wait and see of it comes back. […]’	‘Yes, it has been quite an ordeal and frustrating not to have had the information about the blood clots. I've saved every document I have been given, but no text on blood clot risks and no in-person warning from anyone.’	‘Now that I know better, there won't be any additional shave biopsies in my future! I had no idea going in or I would have requested something different.’
**Healthcare General**			
Bad care/ bad doctors	‘Honestly, I feel like my doctor blows me off a lot too, and after reading this thread I am once again realizing I am my own best advocate.’	‘I had a delay in my diagnosis as my Gp dismissed mine the first time 8 went. .it took 10 months before I got mine removed. I can't help but wish I had insisted much sooner.’	‘ I had to push very hard tor a biopsy and it ended up saving my life. Push for the procedure and find out for certain it's nothing.’
Good care/ good doctors	‘My oncologist thinks that a month of interferon is good enough. He’s been careful in counselling me and watching for depression, which has been only for a few days. His staff is also watchful, too.’	‘My specialist nurse is great I call her with everything! And she really makes me feel at ease and explained everything so well to me she also speeds things up as much as she can, I have two children, mine are 2 & 4, so really I wanted everything over with as quick as possible to get my recovery started!’	‘At the beginning we were sure we'd get a second opinion, but after inhaling as much info as possible, it was clear that treatment would be basically the same anywhere, and our experiences at the center were extremely positive, so there was no need.’

**Health General**			
Diet and appetite	‘He’s also changed his diet (I listened to the audio book Anti-Cancer on my drive to visit, and it had some great suggestions in this regards), and is taking some supplements like modified citrus pectin.’	‘Have you considered the Vit B17 route? I have seen a lot of blogs totally dismissing the idea and yet I have looked into it extensively I believe there could be huge benefits! I guess I also feel anything is worth a shot and some of the testimonies are incredible!’	‘So far I'm doing ok, just not a lot of appetite yet and very tired.‘
Good health	‘pretty good most of the time’	‘I'm very happy to say that I am still feeling fit & healthy & my consultant is very happy with my rude health!’	NA
Pain	‘It took a long time to recover from these surgeries with a lot of nerve pain but after 1 yr I was doing better. For the past 6 wks or so I have had almost non stop pain in my Lt thigh. Feels like nerve pain and is really painful in my 3 and 4 yr old incisions. If I ignore it it become sunbearable and I get a lot of knee pain but usually doesn’t affect my lower leg.’	‘Feeling better in myself, although still rather sore, and now just have to wait. ‘	‘Hurting bone, joints and muscles.... Help HELP: I have been on OPDIVO for 10 treatments so far and 16 more to go, the past two weeks, I have had muscle/bone pain all over, My neck, arms, hands, fingers, hips and legs. Just sitting, I feel so tight but when I move, I just cringe with the aching pain. Anyone else have this issue and what things have helped. I have tired 1000 mg Bayer back and body, little relief. I have also taken 800mg Motrin, again little relief. So, will this go away or is it now with me for the long hall.’
Pain free	NA	‘My arm isn't tight though and it feels very comfortable, with no after pain.’	NA
**Mental Health**			
Depression	NA	‘Hi [user] just to say the blue will fade in time, well mine did!’	NA
Fear/ worry/ anxiety[2]	‘The fear is the worst and we all live with it every day.’	‘I am totally paranoid about being outside uncovered despite having factor 50 on.’	‘This is a roller coaster ride and not the good kind. I remember feeling like I had gotten on an express train and couldn't get off. Once my diagnosis came down I was constantly reeling.[…]’

Mental Health[1]	'[…] yes I am appalled that on some issues I can't seem to get a straight answer. Maybe I ask too many questions??? But that helps me keep sane with this diagnosis!!!'	'I am not sleeping at all well now days, Its not the hot weather and don't think its anything to do with the treatment, it might just be that my head is messed up with everything that all of us on here are going through.'	'The oncologist was happy to announce she considers me NED! She then told me that since I had the brain tumor the last time I was off treatment that we needed to continue with the Opdivo at least another year. Despite the great news I'm hesitant to be too happy and reluctant to consider myself in the clear. I worry that this will not be my last dance with this devil. I'm still bearing the scars from the brain surgery and radiation. My right foot is still numb from the crainiotomy. I'm still not mentally whole.'
No anxiety/ relieve	'I received the biopsy report on the toe mole this past Thursday and it came back benign. I was so relieved (and happy) I wanted to break out in an Irish Jig…despite the painful toe.'	'I have fortunately just had my results this week from my SLNB and WLE which were both clear (hooray)'	'I was extremely relieved at the time because I knew the biopsy report was about as good I could have hoped for, short of it being melanoma in situ.'
Not to worry	'[…] This helped put my mind at ease, as I was really scared.'	'You wonâ€™t believe it but nine months on and I am NOT spending all my waking hours worrying. In fact only when I get my 3 month check does it come to mind. Or occasionally when log onto the forum'	'(One question I always ask myself - if I hadn't had a melanoma diagnosis, would this lump/bump/lesion bother me??? Because if the answer is no, then it's probably still the right answer even after your diagnosis).'
Positive mood	'I had my side ecission in November 2009 and in general things have been healing nicely and I feel wonderful.'	'There is nothing I like more than beating statistics'	'I've been lucky, and have been my own advocate for my care.'
Stress	'I can't sleep or eat. I'm frozen.'	'I too thought dreadful things and physically made myself unwell with the stress and panic.'	'I have struggled to get the replacement doses for my missing hormones correct. I have limited energy and my tolerance for stress had dropped to close to nothing.'

**Physical Health**			
Exercise	‘I have found running to be one of the most helpful things for my lymphedema. Granted, I won’t be running any marathons, but 10k’s are doable and I might even try to work up to a ½ marathon but we’ll see. The key for me is to not overdo it too quickly. It took me a long time to VERY slowly build up to running 5 miles without pusihing too hard.’	‘My son, whom just entering teens, so not very sympathetic said' mum you're walking like an old lady' I'm determined to improve physical health asap especially as spring summer approaches.’	NA
Fatigue	‘Some of the side effects have been a little difficult. Mostly the fatigue. I used to work 10 hour days 5 days per week. That has not been possible, but I’m blessed to work for a supportive company.’	‘Treatment made me tired and it's a bit of a vicious circle, tiredness makes you more inactive and increases tiredness !!’	‘My only complaint has been fatigue. I have tolerated this stuff better than most. I refuse to let the fatigue win.’
Good physically	‘I feel physically good […]’	‘Tell [user] that in my case, the only plus about melanoma (if you could call it that) is apart from the normal after op pains I have never actually suffered physically. Perhaps a bit mentally as it can bombard the senses on a bad day.’	‘[…]My husband feels great and in fact is playing 18 holes of golf today.‘
Pregnancy	‘My new worry is, my husband and I would really like to have another baby, we always wanted 3 kids! But the doctors know I got the Melanoma when I was pregnant with my 2nd son and I am concerned that if I get pregnant again the melanoma will come back or I will get a different type of cancer. I would really like to have another baby but it was soo hard dealing with the cancer and surgery while having a newborn. Do I take the chance or not?’	‘I was pregnant in that time so I didn't bother much about it (I had no idea it can speed the things up). Two months after my baby was born I went to GP and showed him my mole, however 'nothing to worry about.’	‘i have many of the same concerns and am having a tough time wondering if I'll be able to carry another pregnancy. (Daughter is almost 1 yr, she was 7 months when I diagnosed stage 3a).’
**Social Life**			
Family	NA	‘fabulous emotional support from my friends and family. ‘	NA

Friends	NA	‘Thank goodness for my fabulous friends who came round to help me with the babies and to keep me company.‘	‘I had the fortunate experience to have a very dear friend from England, who immediately came to help and stayed with me for 6 months.’
Patient network	‘I cannot emphasize how much I appreciate the support and guidance I have received on this forum. I cannot imagine where I would be right now without it.’	‘its good to share views and feelings on here isn’t it I don’t think anyone who hasn’t gone through any of this can properly understand as I know I didn’t before.‘	‘The encouragement and stories really helped! I'm not much of a poster but check out the site every other day or so. My onc is always amazed at the facts and information I come in with at my appts. It's thanks to all of you.’
Work	‘[…] I’m blessed to work for a supportive company.’	‘I now have secondary adrenal insufficiency and I no longer can cope with the highly stressful parts of my job and the travelling. However, my employers have been great and I am still working three years after a pretty poor prognosis.’	‘So give me the good, bad and ugly about Keytruda. I know everyone reacts differently, but I had no idea what to expect last time and I really would love to know more this time. I have a two year old son and husband, which is different from ten years ago and I am hoping I can still function to work and be a mom.’
**Support**			
Ignorance	‘I can’t tell you how many people, when I tell them I had the melanoma, say, Oh, I had one of those and I just stare at them. Then I say, Really? Where? Did they get it early? And they shrug their shoulders and say, Oh, I don’t really remember. Because, they WOULD remember a melanoma…they HAD some sort of pre-cancer. Argh.’	‘I also have to smile through gritted teeth when I'm told that I'll back to my old self soon etc! No one understands how the lymphatic system works (I didn't until this) so I can never be my old self !‘	‘No one seems to understand how hard it is, especially when im 28!!’
Lack of support	NA	‘Not getting much emotional support from my other half who doesn't really talk just says "it will be fine".’	‘I don't have a big support group because we moved from Texas to Seattle a few years ago.’
Support	‘Thank you all for the feedback and support I have received. I really do appreciate it and don’t know how I would have kept my self sane without having this board to bounce things off of.’	‘but my point is, i sometimes feel very alone, and you guys, although i've never met u offer such great support and comfort at times wen i feel quite lonely, thank you, and wen u all have so much going on too, i think its safe to say there are some remarkable people on this forum.’	‘Rather than get into any more details, I want to just thank those who supported me, and shared their wisdom and experience throughout these years’

**Treatment**			
Drug effectiveness	'I agree that Intron, or interferon, is not the be all, end all of drugs. […]'	NA	'[…] Went to emergency room and they diagnoised with Pneumonia, spent 3 days in hospital with massive IV antibodies. Turns out it is not that but Pneumonitis caused by treatment. At home on oxygen and have appointment on WED. Doc said that it is a reaction to drug. Up until this point I did not have respect for how powerful the drug was, because side effects were minimal.'
Good medicines	'She did the same treatment route that you have and like yourself she has not really had too hard of a time with the Interferon. Other than being tired, she is pretty good most of the time.'	'I had Ipi.I was incredibly lucky in that Ipi worked for me. It activated my T-cells, which destroyed my tumours. I am in the group called 'complete responders'.'	'I'm stage IV and started with the ipi/nivo combo and it worked very well for me. I can't say it was a walk in the park but much better than the treatments for the other cancer types. I'm currently on nivo only and I tolerate it fairly well.'
Randomized Controlled Trials	'I starting to question whether the medical field really care about the people or just the reserach they can do on us.'	'i'm stage 3b and was only offered the avastin trial which i refused cos of theÂ fear of it stopping me being accepted onto any further trials which are more affective down the line.'	'I am afraid they will knock me out of the study but have adrenal failure and hypopituitarism is not healthy at all.'

MIF: Melanoma International Forum; MPIP: Melanoma Patients Information Page; NA: Not Applicable

Figure S1 Number of threads and reply posts of each unique user posted on each forum for the posts assessed

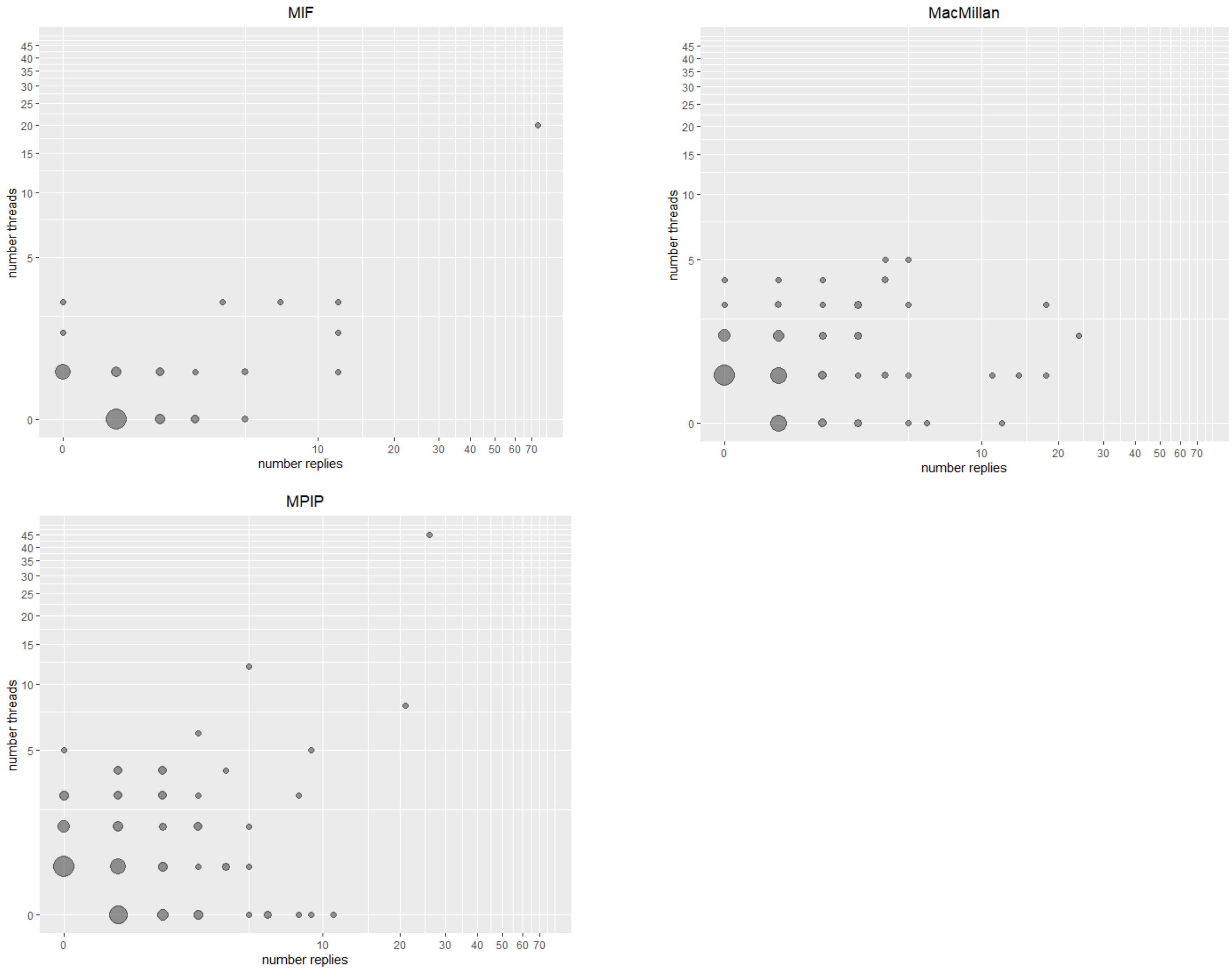

Supplement: Multimedia Appendix 1 [file jmir_v23i12e27497_app1.pdf]
